# Utility of oligonucleotide in upregulating circular RNA production in a cellular model

**DOI:** 10.1038/s41598-024-58663-x

**Published:** 2024-04-06

**Authors:** Lu Ni, Takeshi Yamada, Kazuhiko Nakatani

**Affiliations:** Department of Regulatory Bioorganic Chemistry, SANKEN (The Institute of Scientific and Industrial Research), Osaka University, 8-1 Mihogaoka, Ibaraki, Osaka 567-0047 Japan

**Keywords:** Nucleic acids, RNA

## Abstract

Circular RNAs (circRNAs), are a covalently closed, single-stranded RNA without 5′- and 3′-termini, commonly stem from the exons of precursor mRNAs (pre-mRNAs). They have recently garnered interest, with studies uncovering their pivotal roles in regulating various aspects of cell functions and disease progressions. A notable feature of circRNA lies in the mechanism of its biogenesis involving a specialized form of splicing: back-splicing. A splicing process that relies on interactions between introns flanking the circularizing exon to bring the up and downstream splice sites in proximity through the formation of a prerequisite hairpin structure, allowing the spliceosomes to join the two splice sites together to produce a circular RNA molecule. Based on this mechanism, we explored the feasibility of facilitating the formation of such a prerequisite hairpin structure by utilizing a newly designed oligonucleotide, CircuLarIzation Promoting OligoNucleotide (**CLIP-ON**), to promote the production of circRNA in cells. **CLIP-ON** was designed to hybridize with and physically bridge two distal sequences in the flanking introns of the circularizing exons. The feasibility of **CLIP-ON** was confirmed in HeLa cells using a model pre-mRNA, demonstrating the applicability of **CLIP-ON** as a trans-acting modulator to upregulate the production of circRNAs in a cellular environment.

## Introduction

CircRNAs are covalently closed single-stranded RNA, without a 5′-cap or 3′-polyA tail^1,2^. Though originally thought to be by-products of aberrant splicing^3,4^, circRNAs have garnered significant interest due to their ubiquitous presence across various eukaryotic organisms, tissues, and cell types^5–10^. Recent advances in circRNA research have uncovered their potent regulatory abilities in cells, including acting as miRNA sponges^10–12^, competitive endogenous RNAs for RNA-binding proteins (RBPs)^13^, competitors to canonical splicing^14^, and translation templates^15^. Consequently, circRNA dysregulation has become one of the major focal points in understanding human disease pathologies and progressions, and they have been found to be associated with various diseases, such as cancers and neurological disorders^11,16–19^. In many cases, especially in cancers, the downregulation of certain circRNAs was shown to be a major prognostic factor associated with disease progression, while their upregulation was shown to repress the process^16,17,20^. These characteristic potentials of circRNAs put forth a new challenge to selectively target and modulate specific circRNA expression, and eventually provide a new vector in regulating cellular function. Toward this end, we are attempting to develop such potent regulators by exploiting the biosynthetic mechanism unique to circRNAs: back-splicing.

We previously demonstrated selective upregulation of circRNA production in cells utilizing a small molecule, NCD. The result showed the potential utility of small molecules as an externally introduced trans-regulatory factor to upregulate the production of circRNAs in cells. In the study, NCD facilitated the formation of a prerequisite hairpin structure through the selective binding of a designated target sequence in the flanking introns and upregulated the production of circRNA from a model pre-mRNA^21^. Despite the potency and selectivity shown by such small molecules, finding and screening of suitable compounds for other sequences could be bottlenecks to their application in regulating circRNA production.

**Figure 1:**
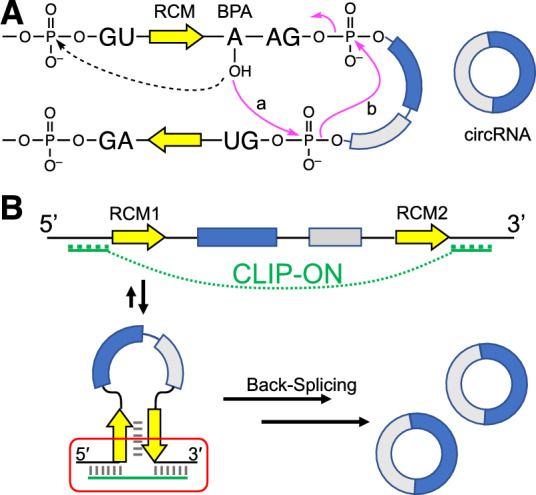
(**A**) Schematic representation of back-splicing (solid pink arrows) to form circRNA and the initial step for the linear splicing (dotted arrow). (**B**) Schematic representation of **CLIP-ON** design bridging sequences flanking the RCM pair (yellow arrows), forming a bridged structure that facilitates the formation of a pre-requisite hairpin structure, and upregulates circRNA production.

In back-splicing, unlike canonical linear splicing for mRNA production, the initial attack of the 2′-hydroxyl group of branch point adenosine (BPA) occurs at the phosphodiester of the 5′-splice site (5′ss) of the downstream intron (Fig. 1A, solid line, step a), as opposed to the upstream 5′ss in linear splicing (Fig. 1A, dotted line). This results in the splicing reaction occurring in the reverse direction (Fig. 1A, solid line), joining the downstream 5′ss with the upstream 3′ss, and the formation of a circRNA^22,23^. To facilitate such a process, the back-splicing reaction commonly requires the presence of inter-intronic interactions between introns flanking the circularizing exons, which is often provided by base pairing between a pair of reverse-complementarily matched sequences (RCM, Fig. 1A, yellow arrows)^24–29^. This results in the formation of a prerequisite hairpin-like structure to bring the BPA and the 5′ss of the downstream intron within close proximity, facilitating the back-splicing reaction. Bioinformatic analysis suggests the presence of distinct complementary repeat sequences often take the role of RCMs, and the duplex stability upon hybridization of RCMs would be a factor that determines the efficiency of circRNA production^25,26^.

Based on the key feature in the back-splicing reaction, the use of oligonucleotides (ONs) was explored as a new trans-acting modality for targeting circRNA upregulation. Owing to the nature of its modality, ONs can be designed in a sequence-dependent manner to target a broad range of RNA sequences, allowing for a fine-tuning of ON binding affinity and functionality via a large library of available nucleotide/sugar/phosphate backbone modifications^30,31^. Here, we demonstrate that **CLIP-ON** promotes the production of circRNA under a model cellular environment. Design-wise, **CLIP-ON** targets two disjointed sequences flanking the circularizing exon(s), physically bridging the flanking introns, creating a new inter-intronic interaction surface, and facilitating the formation of the prerequisite hairpin structure for exon-circularization (Fig. 1B). In a departure from the traditional ON design of targeting continuous adjoining sequences, the approach provides new design concepts for ON designs, allowing the modulation of circRNA production in cells.

## Results

### CLIP-ON promotes circRNA production in model cellular environment and is sensitively dependent on its capacity to bridge distal intronic sequences

**Figure 2:**
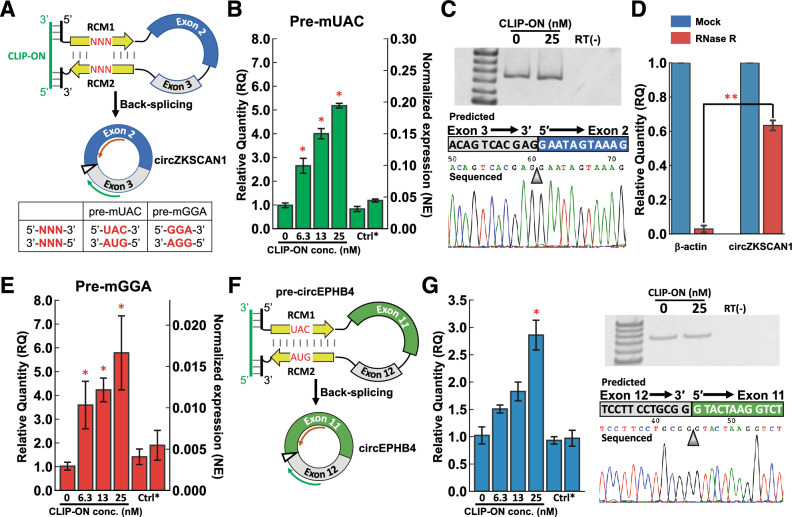
(**A**) Schematic representation of predicted **CLIP-ON**/pre-mRNA complex, followed by the schematic representation of circZKSCAN1. The site of the back-splice junction is shown with a triangle, while the green and orange arrows represent the primers used for the qPCR. The table denotes the nucleotide sequences modified between **pre-mUAC** and **pre-mGGA**. (**B**) **CLIP-ON** concentration-dependent change of circZKSCAN1 in HeLa cells transfected with ***p-*UAC**, expressing **pre-mUAC** (n = 4). (**C**) PAGE analysis of amplified qPCR products (nontreated, 25 nM **CLIP-ON** treated, and non-RT), and the typical sequencing result of the amplified PCR product. (**D**) qPCR results following RNase R treatment of total RNA obtained from **pre-mUAC** expressing HeLa cells (n = 3). (**E**) **CLIP-ON** concentration-dependent circZKSCAN1 change for HeLa cells transfected with ***p-*GGA**, expressing **pre-mGGA** (n = 4). (**F**) Schematic representation of pre-mRNA modified to include exon 11 and 12 from gene EPHB4, **pre-circEPHB4**, and resulting circRNA, circEPHB4. (**G**) **CLIP-ON** concentration-dependent circEPHB4 change for HeLa cells transfected with *p*-circEPHB4, expressing **pre-circEPHB4** (n = 4), followed by PAGE analysis of amplified qPCR products (nontreated, 25 nM **CLIP-ON** treated, and non-RT), and the typical sequencing result of the amplified PCR product. The original gels of (C) and (G) are presented in Sup. Fig. 2. Full sequencing data for (C) and (G) are presented in Sup. Fig. 3 (Ctrl*: 25 nM **Scram-ON** (left) and **part-CLIP-ON** (right) treated samples; *: *p* < 0.005, RQ > 2; the significance was obtained against the sample treated with 0 µM **CLIP-ON**, using two-tailed t-test; **: *p* < 10^–5^, two-tailed t-test).

The efficacy of **CLIP-ON** as an externally introduced trans-factor in promoting circRNA production was examined in HeLa cells utilizing a pre-mRNA model, previously reported by Wilusz et al.^25^. Briefly, the pre-mRNA consists of exons 2 and 3 of gene ZKSCAN1, flanked by a pair of 36-nt RCMs (Fig. 2A, Sup. Figure 1A, B), which has been shown to produce a well-studied circZKSCAN1 via an RCM-facilitated back-splicing pathway. To differentiate from a modified pre-mRNA design used later in the article, where a trinucleotide mismatch was introduced into the RCM, the plasmid was named ***p-*UAC** based on the nucleotide difference and transcribed pre-mRNA as **pre-mUAC**. As for **CLIP-ON**, to ensure binding, the upper end of the typical ON design length, 25-nt, was used^32^. The design involves two 12-nt segments targeting the sequences flanking the above-mentioned RCMs, which were then bridged by a 1-nt uracil linker as a spacer between the two independent hybridizing sections. The sequence selection was further optimized to minimize extended self-complementarity and fulfill the standard requirements of ON designs, where the predicted energy was > –4 kcal mol^−1^ for ON and > –15 kcal mol^−1^ for ON-ON complexes^33^. **CLIP-ON** was then further fully modified with a phosphorothioate backbone and 2′-*O*-methyl, to improve its stability in the cellular environment^30,31^. In addition, as control ONs, a partially hybridizing control (**part-CLIP-ON**) and a fully scrambled control (**Scram-ON**) were utilized. All ON sequences are listed in Table 1. The changes in circZKSCAN1 production from the model pre-mRNA, **pre-mUAC**, following treatment with various **CLIP-ON** in cells were assessed using RT-qPCR. The relative quantity (RQ) of circZKSCAN1 in cells treated with **CLIP-ON** compared with untreated cells (**CLIP-ON** 0 nM treated cells) was then obtained by using the comparative Ct (ΔΔCt) method^34,35^. The “divergent” primer utilized specifically to amplify the circZKSCAN1 stemming from the model was designed as described previously (Fig. 2A, Sup. Figure 1B)^1,21,35^. Where a single nucleotide difference in exon 3 of plasmid-expressed circZKSCAN1 was utilized to allow the discrimination from endogenous circZKSCAN1 via primer design^36^.

**Table 1:** Oligonucleotide sequences used in experiments, the linker uracil is highlighted in bold.

Oligonucleotide name	Sequence
CLIP-ON	5′-GACUCGAGCGGC **U** CUUUGAAUUCCA-3′
CLIP-ON-10	5′-CUCGAGCGGC **U** CUUUGAAUUC-3′
CLIP-ON-8	5′-CGAGCGGC **U** CUUUGAAU-3′
CLIP-ON-NoLink	5′-GACUCGAGCGGC CUUUGAAUUCCA-3′
CLIP-ON-3Link	5′-GACUCGAGCGGC **UUU** CUUUGAAUUCCA-3′
Scram-ON	5′-AUGAUUCCUUCGAGAGCUCCGCGAU-3′
Part-CLIP-ON	5′-GACUCGAGCGGCAUCUCUCUGUAUA-3′

All ONs were fully 2′-*O*-methyl phosphorothioate modified.

Upon treatment with **CLIP-ON**, HeLa cells expressing the model pre-mRNA (**pre-mUAC**) showed an increase in the expression of circZKSCAN1 in a **CLIP-ON** concentration-dependent manner. Compared with untreated samples, cells treated with **CLIP-ON** achieved up to an apparent 5.2-fold (*p* < 5 × 10^–8^), 4.0-fold (*p* < 5 × 10^–7^), and 2.6-fold (*p* < 5 × 10^–4^) increase in circZKSCAN1 expression at the treated **CLIP-ON** concentration of 25, 13, and 6.3 nM, respectively. However, no statistically significant changes were observed for cells treated with control ONs, **part-CLIP-ON**, or **Scram-ON**, under the same 25 nM concentration (Fig. 2B). The qPCR amplified target and primer specificity was further confirmed via PAGE analysis followed by sequencing, confirming the presence of the back-spliced junction sequence stemming from circZKSCAN1 and the circularity was then further confirmed by its resistance to RNase R treatment relative to the linear mRNA of β-actin (Fig. 2C, D).

### CLIP-ON can promote circRNA production from pre-mRNA with destabilized RCM interaction and different exons

To clarify whether bridging by **CLIP-ON** requires the distal target sites in the introns to be prearranged by stable RCM pairing, a 5′-GGA-3′/5′-GGA-3′ trinucleotide mismatch was introduced into the RCMs (Fig. 2A, Sup. Figure 1A, C, the plasmid hereon as ***p-*GGA** and transcribed pre-mRNA as **pre-mGGA**), as the introduced mismatch site was previously shown to destabilize the hybridization of RCMs and impair the production of circZKSCAN1. Additionally, β-actin normalized expression (2^–ΔCt^, hereon as NE) was also calculated in addition to RQ to allow cross-examination of the expression between the two model pre-mRNAs, **pre-mUAC** and **pre-mGGA**, following ON treatment.

Upon treatment with **CLIP-ON**, HeLa cells expressing the **pre-mGGA** showed **CLIP-ON** concentration-dependent increase in the expression of circZKSCAN1 similar to that of **pre-mUAC**. Compared with untreated samples, cells treated with **CLIP-ON** achieved up to an apparent 5.8-fold (*p* < 5 × 10^–3^), 4.2-fold (*p* < 5 × 10^–5^), and 3.6-fold (*p* < 5 × 10^–3^) increase in circZKSCAN1 expression at the treated **CLIP-ON** concentration of 25, 13, and 6.3 nM, respectively, and no statistically significant changes were observed for cells treated with control ONs under the same 25 nM concentration (Fig. 2E). Despite similar fold increase in upregulation, the normalized expression of circZKSCAN1 in HeLa cells expressing **pre-mGGA** was 13-fold lower (NE = 0.0028) than that of **pre-mUAC** (NE = 0.037), in untreated samples (Fig. 2B, E). The difference was reduced to twofold (NE = 0.016) lower for **pre-mGGA** upon treatment with 25 nM **CLIP-ON**, compared to untreated **pre-mUAC** samples.

To determine the effects of exon on the efficacy of **CLIP-ON**, the ZKSCAN1 exons were also replaced by exons that comprise circEPHB4, exons 11 and 12 from gene EPHB4 and were modified to allow discrimination from endogenous circEPHB4 in a similar manner to circZKSCAN1. The divergent primers were then designed with an additional mismatch in the third position from the 3’ end of the reverse primer for specific amplification of the desired segment, (Fig. 2F, Sup. Fig. 4, the plasmid hereon as ***p*-circEPHB4** and transcribed pre-mRNA as **pre-circEPHB4**). Upon treatment, **CLIP-ON** similarly showed the ability to concentration-dependently upregulate the expression of circEPHB4, achieving a 2.9-fold (*p* < 5 × 10^–4^) increase in the production of circEPHB4 compared with untreated samples. Again, no statistically significant changes were observed for cells treated with control ONs under the same 25 nM concentration (Fig. 2G). The qPCR amplified target and primer specificity was further confirmed via PAGE analysis followed by sequencing, confirming the presence of the back-spliced junction sequence stemming from circEPHB4 and the circularity was further confirmed by its resistance to RNase R treatment (Fig. 2G, Sup. Fig. 5).

### In vitro experiments confirm the binding of CLIP-ON and the capacity to bridge distal introns

**Figure 3:**
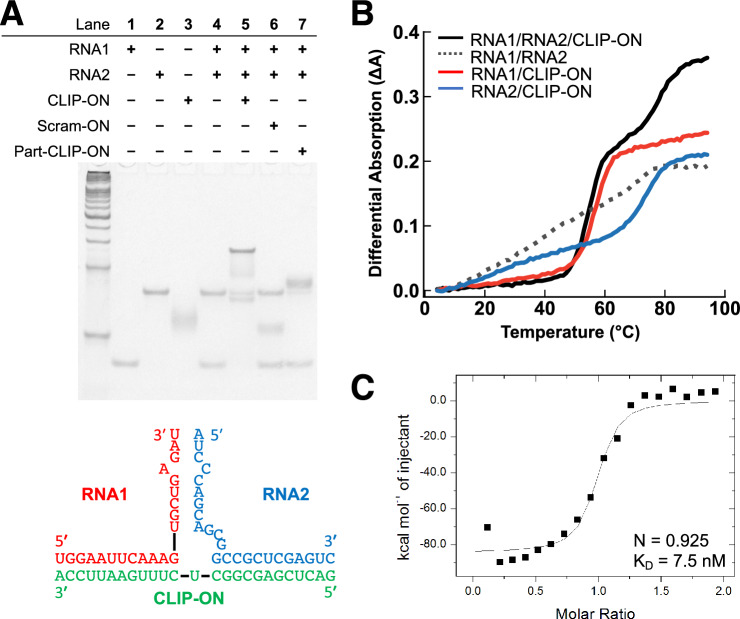
(**A**) Native PAGE results obtained when various RNA and/or ONs mixed under equimolar conditions. The table (top) lists the components which were loaded in each lane. The predicted hybridized structure and the sequence of RNA1/RNA2 used for the native PAGE are shown (bottom). For the size marker, 20 bp DNA ladder was used. (**B**) The representative thermal melting (*T*_m_) profile of RNA1/RNA2/**CLIP-ON** mixture (black line) compared to RNA1/RNA2 (dotted line), RNA1/**CLIP-ON** (red line), and RNA2/**CLIP-ON** (blue line). (**C**) ITC analysis of **CLIP-ON** and target RNA sequence hybridization. The original gels of (**A**) in Sup. Fig. 7.

The hybridization between **CLIP-ON** and the two distal intronic target sites was first determined via native polyacrylamide gel-electrophoresis (native PAGE). An equimolar concentration of partial RNA sequence of **pre-mUAC** (RNA1 and RNA2) and ONs, **CLIP-ON**, **part-CLIP-ON**, and **Scram-ON** was used. In the absence of **CLIP-ON** (Fig. 3A lane 4), two distinct bands were observed corresponding to the respective RNAs (Fig. 3A lanes 1 and 2), with RNA2 having significantly lower mobility than RNA1 due to RNA2 having a high GC content and potentially form stable self-dimers (Sup. Fig.  6); by itself, **CLIP-ON** showed a smeared band (Fig. 3A lane 3). Although RNAs by themselves showed no hybridization under PAGE conditions (Fig. 3A lane 4), upon addition of **CLIP-ON**, a new low-mobility band was observed in addition to the depletion of faster mobility bands corresponding to RNA1, RNA2, and **CLIP-ON** (Fig. 3A lane 5), while a small fraction of band corresponding to remaining uncomplexed RNA2 dimer and RNA1/**CLIP-ON** was also observed. By contrast, **Scram-ON** showed distinct bands corresponding to the two RNAs and **Scram-ON** (Fig. 3A lane 6), whereas **part-CLIP-ON** showed a slight shift in the RNA2 band associated with **part-CLIP-ON**/RNA2 hybridization (Fig. 3A lane 7) and no changes were observed for RNA1.

The thermal stability of the hybridized structure was determined via thermal melting measurements (*T*_m_) (Fig. 3B). In the presence of an equimolar concentration of the two RNAs and **CLIP-ON**, a biphasic sigmoidal curve in the *T*_m_ profile was observed, *T*_m1_ = 53.7 °C (S.D. = 0.9 °C) and *T*_m2_ = 75.8 °C (S.D. = 1.8 °C), respectively. No apparent *T*_m_ was observed in the absence of **CLIP-ON** (Table 2). The two observed *T*_m_ values for the RNA1/RNA2/**CLIP-ON** mixture were shown to correspond to the *T*_m_ of RNA1/**CLIP-ON** (56.5 °C) and RNA2/**CLIP-ON** (72.8 °C) when they were measured independently. However, in the RNA1/RNA2/**CLIP-ON** complex, the corresponding dissociation temperature showed deviation from the independently measured *T*_m_ of RNA1/**CLIP-ON** and RNA2/**CLIP-ON** by –2.8 °C and + 3.0 °C, respectively, indicating the presence of cooperativity in the formation of the RNA1/RNA2/**CLIP-ON** complex. This was further evaluated by the largest tangent angle (TA) of the UV-melting profiles. TA corresponds to the slope of the sigmoidal curve at the *T*_m_ value, for which larger values indicate more abrupt dissociation of duplex, which is indicative of higher cooperativity^37,38^. The TA corresponding to the first dissociation of the RNA1/RNA2/**CLIP-ON** complex, *T*_m1_ (0.021 °C^–1^), is larger than that of RNA1/**CLIP-ON** (0.016 °C^–1^), whereas TA corresponding to the second dissociation, *T*_m2_ (0.008 °C^–1^), remained unchanged from that of RNA2/**CLIP-ON** (0.008 °C^–1^).

**Table 2:** Calculated Tm and TA values for the thermal melting profile in Fig. 3B.

Sample	T_m1_ (°C)	S.D.	T_m2_ (°C)	S.D.	TA_1_ (°C^–1^ )	TA_2_ (°C^–1^ )
RNA1/RNA2/**CLIP-ON**	53.7	0.9	75.8	1.8	0.021	0.008
RNA1/RNA2	–	–	–	–	–	–
RNA1/**CLIP-ON**	56.5	–	–	–	0.016	–
RNA2/**CLIP-ON**	–	–	72.8	–	–	0.008

*S.D*. standard deviation.

The apparent binding affinity of **CLIP-ON** toward its target RNA sequences was further clarified via isothermal titration calorimetry (ITC, Fig. 3C). An RNA comprised of partial RCM sequence and the relevant flanking sequences joined by a 4-uracil linker was used to simulate a 1:1 binding of **CLIP-ON** toward the target pre-mRNA site (RNA3, sequence listed in Table S1). The results show **CLIP-ON** binding to the RNA target with an equilibrium dissociation constant (*K*_*D*_) of 7.5 nM. The obtained *K*_*D*_ was further confirmed with a surface plasmon resonance (SPR) assay, which showed a *K*_D_ of 10 nM (Sup. Fig. 8).

### Thermal stability of CLIP-ON/RNA complex dictates CLIP-ON activity

**Figure 4:**
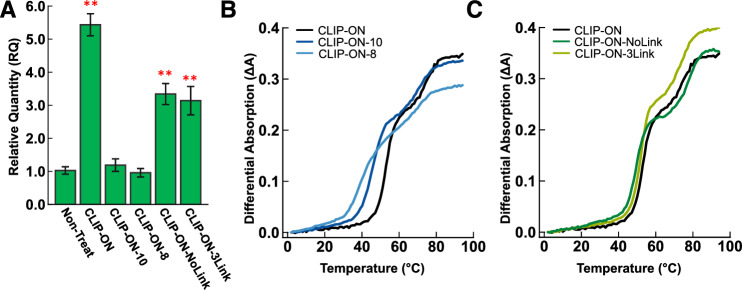
(**A**) **CLIP-ON** variant dependent change in expression of circZKSCAN1 in HeLa cells expressing **pre-mUAC**, in all cases HeLa cells were treated with 25 nM of the respective ONs. (**B**) The representative *T*_m_ profile of **CLIP-ON** variants with truncated hybridizing segments, **CLIP-ON-10** (dark blue line) and **CLIP-ON-8** (light blue line), with RNA1 and RNA2. (**C**) The representative *T*_m_ profile of **CLIP-ON** variants with modified uracil linker, **CLIP-ON-NoLink** (dark green line) and **CLIP-ON-3Link** (light green line), with RNA1 and RNA2. The calculated respective *T*_m_ values for both (B) and (C) are shown in the table below, while the standard deviation (S.D.) is listed within brackets. (**: *p* < 5 × 10^–4^, RQ > 2; the significance was obtained against the sample treated with 0 µM **CLIP-ON**, using two-tailed t-test, n = 4).

A variation of **CLIP-ON** with either 10-nt or 8-nt segments (**CLIP-ON-10** and **CLIP-ON-8**, respectively), or with 0-nt or 3-nt uracil linker (**CLIP-ON-NoLink** and **CLIP-ON-3Link**, respectively) was also prepared to further elucidate the effects of various components on the viability of **CLIP-ON** design. Towards this end, HeLa cells expressing **pre-mUAC** were treated with 25 nM of the above variants of **CLIP-ON**, and the changes in RQ of circZKSCAN1 were then determined using qPCR (Fig. 4A). The results show that for **CLIP-ON** variants with reduced hybridizing segment length, **CLIP-ON-10** and **CLIP-ON-8**, the ability to induce upregulation in circZKSCAN1 was completely abolished in both cases. On the other hand, despite having the same hybridizing segment as the original design **CLIP-ON** variants with different linker lengths, **CLIP-ON-NoLink** and **CLIP-ON-3Link**, showed a reduced biological activity, providing an apparent 3.3-fold (*p* < 5 × 10^–5^) and 3.1-fold (*p* < 5 × 10^–4^) upregulation of circZKSCAN1 at 25 nM, respectively.

To elucidate the underlying factors behind the observed differences in biological activity between the variants of **CLIP-ON**, a thermal melting analysis was conducted. For **CLIP-ON** variants with reduced hybridizing segments, the thermal melting profile corresponding to the dissociation of the bridged structure (*T*_m1_) was to 45.6 °C (S.D. = 0.1 °C) and 40.0 °C (S.D. = 0.4 °C) for **CLIP-ON-10** and **CLIP-ON-8**, respectively (Fig. 4B, Table 3). Interestingly, despite having the same hybridizing segment length, **CLIP-ON** variants with different linker lengths also showed a decrease in *T*_m1_, with 49.5 °C (S.D. = 0.1 °C) and 51.8 °C (S.D. = 0.3 °C) for **CLIP-ON-NoLink** and **CLIP-ON-3Link**, respectively (Fig. 4C, Table 3). To elucidate the reason behind the observed difference in *T*_m_ between **CLIP-ON** variants with differing linker lengths, their binding characteristics were assessed using ITC (Sup. Fig. 9). The results show that these **CLIP-ON** variants bind with a *K*_*D*_ of similar order and free energy (*ΔG*) as the **CLIP-ON** design (Table 4). Where *K*_D_ was 8.7 nM and 2.8 nM for **CLIP-ON-NoLink** and **CLIP-ON-3Link** respectively; while calculated *ΔG* was –11.1 kcal mol^–1^, –11.0 kcal mol^–1^, and –11.7 kcal mol^–1^ for **CLIP-ON**, **CLIP-ON-NoLink**, and **CLIP-ON-3Link** respectively. However, further examination showed a shift in the thermodynamic parameters of hybridization upon changing the linker length of **CLIP-ON**. Relative to the **CLIP-ON** design, which showed *ΔH* = –84.1 kcal mol^–1^ with *ΔS* = –245 cal mol^–1^ °C^–1^, **CLIP-ON-NoLink** binding was more enthalpically favorable with *ΔH* = –93.1 kcal mol^–1^ but entropically less favorable with *ΔS* = –275 cal mol^–1^ °C^–1^; on the other hand, **CLIP-ON-3Link** binding was less enthalpically favorable with *ΔH* = –71.3 kcal mol^–1^ but was compensated by being more entropically favorable with *ΔS* = –200 cal mol^–1^ °C^–1^ (Table 4).

**Table 3:** Calculated T_m1_ and TA values for the thermal melting profile in Fig. 4B, C.

Oligonucleotide	T_m1_ (°C)	S.D
CLIP-ON	53.7	0.9
CLIP-ON-10	45.6	0.1
CLIP-ON-8	40.1	0.4
CLIP-ON-NoLink	49.5	0.1
CLIP-ON-3Link	51.8	0.3

*S.D*. standard deviation.

**Table 4:** The thermodynamic parameters obtained from ITC measurements for **CLIP-ON** variants with different linker lengths.

Oligonucleotide	K_D_ (nM)	*ΔG* (kcal mol^–1^)	*ΔH* (kcal mol^–1^)	*ΔS* (cal mol^–1^ °C^–1^)
CLIP-ON	7.5	− 11.1	− 84.1	− 245
CLIP-ON-NoLink	8.7	− 11.0	− 93.1	− 275
CLIP-ON-3Link	2.8	− 11.7	− 71.3	− 200

## Discussion

The cellular experiments showed that **CLIP-ON** can upregulate the production of circZKSCAN1 up to 5.2-fold in a concentration-dependent manner, using HeLa cells expressing the model pre-mRNA, **pre-mUAC** (Fig. 2B). No changes were observed when cells were treated with partial and fully scrambled sequences. These observations suggested that the physical bridging induced by **CLIP-ON** played a crucial role in facilitating the formation of the prerequisite hairpin structure to promote the production of circZKSCAN1 from the model pre-mRNAs. In experiments using another model pre-mRNA with destabilized RCM, **pre-mGGA**, circZKSCAN1 was similarly upregulated 5.8-fold at the highest treated **CLIP-ON** concentration (Fig. 2E). Though overall expression was lower for cells expressing **pre-mGGA** compared to **pre-mUAC**, due to the lower stability of RCM hybridization in **pre-mGGA**^21,25^, at the highest treated concentration, **CLIP-ON** showed the ability to compensate for the reduced stability and rescue the expression of circZKSCAN1 up to 43% of HeLa cells expressing the full match counterpart. Demonstrating **CLIP-ON**’s capacity to bind and compensate for the loss in RCM hybridization stability introduced by the mismatches, indicating that the stable hybridization of RCMs is not a necessary requirement for **CLIP-ON**’s biological activity. Experiments using pre-mRNAs containing exons of other circRNA, **pre-circEPHB4**, **CLIP-ON** showed the capacity to circularize exons other than ones comprising circZKSCAN1, albeit with differing efficiencies, achieving a 2.9-fold upregulation of circEPHB4 (Fig. 2G). The results suggest that the ability of **CLIP-ON** to upregulate circRNAs in the cellular environment is independent of the exon in question.

To confirm **CLIP-ON**’s observed mode of action, in vitro experiments were conducted. The native gel-electrophoresis results (Fig. 3A) show that the introduction of **CLIP-ON** induced the formation of a new low-mobility band in addition to the depletion of RNA bands associated with the partial sequence of the target model pre-mRNA. In contrast, the introduction of control ONs, **part-CLIP-ON** and **Scram-ON**, showed no such shift in the band mobility of RNAs. The results indicated the successful formation of the RNA1/RNA2/**CLIP-ON** bridged structure and further confirmed the validity of the **CLIP-ON** design concept and its ability to hybridize and facilitate interaction between two sequences that are located spatially distant from each other. The stability of such a bridged structure was then analyzed by thermal melting analysis (Fig. 3B, Table 2). The observed *T*_m1_ of 53.7 °C (S.D. = 0.9 °C), corresponding to the dissociation of one of the RNA strands, suggests the complex has a very high thermal stability and that the equilibrium strongly favors the formation of the desired bridged structure at physiological temperature. This was further confirmed by ITC and SPR measurements, which showed a low-nanomolar range for the equilibrium dissociation constant for the complex formation (Fig. 3C, Sup. Fig. 8). Although the appearance of two distinct meltings was attributed to the step-wise dissociation of the RNA strands from **CLIP-ON** in an independent manner. The observed shift in *T*_m_ values corresponding to the dissociation of each RNA strand from the RNA1/RNA2/**CLIP-ON** complex indicated the presence of cooperativity of complex formation. This was further clarified using TA, where the slope of *T*_m1_ (0.021 °C^–1^), associated with the initial stage of complex dissociation, was larger than the corresponding TA of RNA1/**CLIP-ON** (0.016 °C^–1^) (Table 2), suggesting that there was an increase in cooperativity upon formation of the bridged complex. The in vitro results indicated that the designed **CLIP-ON** could bind to two distal sequences with high affinity and thermal stability, supporting the observation that the formation of the stable ternary complex, bridged structure, induced the upregulation of circRNAs in cellular experiments.

To further elucidate the relation between the various design features of **CLIP-ON**, hybridizing segment and linker, on the biological activity of **CLIP-ON**, cellular experiments were conducted using variants of the **CLIP-ON** design. **CLIP-ON** variants with truncated hybridizing segments were found to result in the complete loss of circRNA promoting activity (Fig. 4A). These observations were supported by the large reduction in the melting profile of the said **CLIP-ON** variants, where an 8.1 °C decrease in melting temperature was observed upon truncation of 2-nt from the **CLIP-ON**’s hybridizing segment (Table 3), indicating the major role of the thermal stability of the bridging complex in promoting circRNA production. On the other hand, the selection of uracil linker length was also shown to impact the biological activity and thermostability of the **CLIP-ON**. Modifying the uracil linker length of the **CLIP-ON** resulted in a reduction of fold upregulation of circRNA expression (Fig. 4A), and up to 4.2 °C reduction in the melting temperature of the resulting bridging complex was observed (Fig. 4C, Table 3). Further examination using ITC expectedly revealed a similar *K*_D_ and *ΔG* of binding between all three **CLIP-ON** variants, however, a change in the contributing thermodynamic parameters,* ΔH* and *ΔS*, was revealed upon modification of the linker length (Table 4). These results suggest that despite not actively contributing to the hybridization and formation of the bridging structure, the linker length likely plays a crucial role in determining the stability of the resulting complex by modulating the spatial arrangement of hybridizing segments and relaxation of rotational strain involved in hybridization with the two non-continuous distal sequences. These results further revealed that the biological activity of **CLIP-ON** was sensitively dependent on the thermal stability of the resulting bridging structure, in which both the hybridizing segment and the linker selection play a major role.

Overall, based on the obtained results showing the upregulation of circRNA in a cellular environment, the formation of bridged ternary structure in vitro, and the sensitive dependence of biological activity on the thermal stability of the formed bridged structure, we have demonstrated that our new ON design, **CLIP-ON**, can upregulate circRNA production in a cellular model likely in accordance with the hypothesized mechanism. These results further demonstrate the applicability of externally introduced trans-acting modulators in upregulating circRNA production in a cellular environment and present a stepping-stone in molecular designs that regulate circRNA production. It provides interesting prospects for future ON designs, as it demonstrates the utility of modified ONs for targeting distal sequences to promote or invoke biologically relevant secondary structures in a cellular environment, representing a departure from traditional ON designs.

Amidst the increasing evidence of the involvement of circRNA dysregulation in diseases and the potential therapeutic utility of circRNA is currently extensively studied^40^. Currently, transfection of circRNA expression vectors via adeno-associated virus is at the forefront as the potential candidate for such application^41^. Here, the results of this study offer a new strategy to utilize ONs to directly modulate the expression of specific circRNAs. Allowing potential organ-specific targeting through direct chemical modification of the ONs^42^, and side-step potential unwanted mis-spliced side products^40^ and inherent risk of insertional mutagenesis arising from utilizing virus-associated vectors^43^. Future developments in the elucidation of pre-mRNA secondary structures, the flexibility of ON in target selection and designs could be fully leveraged to offer potential new strategies to regulate such biologically relevant circRNAs.

## Material and methods

### General information

Surface plasmon resonance (SPR) measurements were conducted using BIAcore T200 instrument (GE Healthcare). *T*_m_ melting temperature measurements were conducted on UV-2700 UV–Vis spectrophotometer (SHIMADZU). ITC was conducted using MicroCal iTC200 calorimeter (Malvern Panalytical). The original plasmid construct, ***p*-UAC**, used in these experiments: pcDNA3.1( +) ZKSCAN1 nt 400–1782 delta440-500 delta1449-1735, was a gift from Jeremy Wilusz (Addgene plasmid # 60633 ; http://n2t.net/addgene:60633 ; RRID:Addgene_60633)^24^ and ***p-*GGA** was prepared as described previously^21^. HeLa genomic DNA was purchased from New England Biolabs Inc. and plasmid construct ***p*-circEPHB4** was prepared from ***p*-UAC** using in-Fusion^®^ Snap Assembly Master Mix. Escherichia coli (*E.* *coli*) strain DH5α was used to amplify plasmid DNA and the amplified plasmid DNA was extracted and purified using NucleoBond^®^ Xtra Midi EF (Macherey–Nagel). HeLa cells (RIKEN BRC, RCB0007) were used for transfection and were maintained at 37 °C under 5% CO_2_ in Dulbecco’s modified eagle’s medium (Sigma, D6429) supplemented with 10% (v/v) fetal bovine serum (MP Biomedicals) and penicillin–streptomycin (Gibco). All primers were purchased from Invitrogen. All 2′-omethyl phosphorothioate modified oligonucleotides (2′-OMe PS ON) were purchased from GeneDesign Inc. The feasibility of ON designs was assessed and predicted RNA structures were visualized using ViennaRNA package 2.6.1^39^. FuGENE HD™ transfection reagent (Promega) was used for plasmid transfection and Lipofectamine 3000 (Thermo Fisher Scientific) was used for ON transfection. For RT-qPCR experiments, cell lysis and reverse transcription were conducted using Superprep^®^ II cell lysis & RT kit (TAKARA), and real-time PCR (qPCR) experiments were conducted using QuantStudio™ 1 system (Applied Biosystem) using GoTaq^®^ qPCR master mix (Promega). For Ribonuclease R (RNase R) treatment, cell lysis and total RNA extraction were conducted using NucleoSpin RNA (TAKARA), RNase R was purchased from Epicenter Technologies, and the reverse transcription was conducted using ReverTra Ace^®^ qPCR RT Master Mix with gDNA remover (TOYOBO).

### SPR assay

#### (1) Sensor preparation

For the SPR assay, RNA containing the partial intronic sequence containing the **CLIP-ON** target sites was immobilized onto the sensor chip SA (Cytiva), where the surface was coated with streptavidin. The surface of the sensor chip was first washed three times with 50 mM NaOH and 1 M NaCl for 60 s with a flow rate of 30 μL min^−1^. Then the biotin-labeled RNAs, biotin-TEG-5′-UGGAAUUCAAAGUGCUGAGAUUACAGGCGU GAGCUUUUGCUCACACCUGUAAUCCCAGCAGCGGCCGCUCGAGUC-3′ (b-TEG-RNA3, The **CLIP-ON** target regions are underlined), were immobilized onto the surface under the following conditions: 0.5 μM RNA in 10 mM HEPES, 500 mM NaCl, pH 7.4, the flow rate of 5 μL min^−1^ for the flow of 60 s. The amount of RNA immobilized on the sensor chip SA was 990 RU.

#### (2) SPR analysis protocol

SPR analysis for the binding of **CLIP-ON** to the RNA-immobilized surface was conducted on BIAcore T-200 and carried out by subsequently flowing 62.5,125, 250, 500, and 1000 nM of each compound for 60 s of contacting time in HBS-EP + buffer (10 mM HEPES, 150 mM NaCl, 3 mM EDTA, 0.05% v/v Surfactant P20, pH 7.4) (Cytiva) with the flow rate of 30 μL min^−1^ at 25 °C followed by dissociation of bound compounds by flowing running buffer for 120 s. Surface regeneration was carried out after the assay of each compound using 1.2 mM NaOH, 0.2 M NaCl, and 0.1 mM EDTA solution, with a contact time of 180 s and a flow rate of 30 μL min^−1^. Kinetic parameters of the binding of **CLIP-ON** to the RNA-immobilized surface were obtained by using the single-cycle kinetics method.

### Reverse-transcription quantitative PCR (RT-qPCR experiments)

The HeLa cells were plated in a 96-well plate at a density of 1 × 10^4^ cells/well and were incubated for 4 h to allow the cells to adhere to the well bottom. It was then transfected with either ***p-*UAC**, ***p-*GGA**, or ***p*-circEPHB4** expressing plasmid using Fugene HD™ with the ratio (FuGENE : plasmid = 5:2, v/w) according to the manufacturer’s instructions. After 24 h incubation under 37 °C with 5% CO_2_, the medium was exchanged with fresh medium, before being transfected with ONs using Lipofectamine 3000, according to the manufacturer’s instructions, and the cells were further incubated for 24 h. The transfected cells were lysed and the total RNA was reverse transcribed into cDNA using the Superprep^®^ II cell lysis & rt kit according to the manufacturer’s instructions. The resulting cDNA was diluted twofold before performing qPCR analysis.

qPCR experiments were conducted with QuantStudio™ 1 system (Applied Biosystem) and GoTaq^®^ qPCR master mix (Promega) using the cDNA as the template. Each 10 μl reaction contained 1 μl of the cDNA solution, 0.2 μl each of forward and reverse primers (10 μM), 0.1 μl of 100 × CXR dye, and 5 μl of GoTaq^®^ qPCR Master Mix. A negative control without cDNA template or samples without reverse transcriptase was included in each assay. The primers used for the detection of β-actin and the various target circRNAs are listed below in Table S1.

For comparison of the fold change in expression from cells transfected with the same plasmid under various concentrations of **CLIP-ON**, the relative quantity (RQ) was obtained using the comparative Ct (ΔΔCt) method by subtracting the cycle threshold value (C_t_) of target circRNA twice, first from that of β-actin then from target circRNA of untreated sample. The RQ was then obtained accordingly through RQ = 2^−^^ΔΔCt^^34^. To compare the relative difference in circZKSCAN1 upregulation between **pre-mUAC** and **pre-mGGA**, the relative expression of circZKSCAN1 to the endogenous control β-actin was used. For which the cycle threshold value (C_t_) of circZKSCAN1 was first subtracted from that of β-actin, and normalized expression value (NE) was then obtained through NE = 2^−^^ΔCt^. The qPCR products were analyzed by 8% (29:1, acylamide/bis-acrylamide) polyacrylamide gel electrophoresis to check the amplicon size. The specificity of primer pairs was also verified with the presence of a single peak in the melting curve after PCR amplification and sequencing of the amplicon.

For RNase R treatment, HeLa cells plated in a 24-well plate (5 × 10^4^ cells/well) were first transfected with the expression plasmid and treated in the same manner as described above. Total RNA was extracted using NucleoSpin RNA according to the manufacturer’s instructions. 500 ng of the total RNA was then incubated for 10 min at 37 °C with or without 1 U/μg RNase R. The resulting solution was directly subjected to reverse transcription using ReverTra Ace^®^ qPCR RT Master Mix with gDNA remover, according to manufacturer’s instructions. The expression was then analyzed by qPCR as mentioned above, the experiment was conducted 3 independent times.

### Isothermal titration calorimetry (ITC) assay

A solution of hairpin RNA containing partial RCM and **CLIP-ON** binding sequence (RNA3, 1 µM) was titrated with a solution containing **CLIP-ON** and its variants (10 µM) at 25 °C cell temperature in sodium phosphate buffer (10 mM, pH 7.0) containing NaCl (100 mM). The temperature inside the cell was kept at 25 °C during the experiment. The titration experiment was performed by 19 times injections of the titrant (0.4 µL for the first and 2 µL for all following injections) from the syringe into the sample cell at 25 °C (stirred at 750 rpm). The initial time prior to the first injection was 60 s. The duration for each injection was 4 s, and the interval between the two nearest injections was 150 s. Additionally, the titrant was also injected into the cell containing only buffer solution in the same manner to obtain the data for the heat of dilution of the titrant. Prior to fitting analysis, the data point corresponding to the first injection was removed, and the heat data were corrected by subtracting the heat of dilution. Binding isotherms were fitted to the Origin models by least-squares analysis.

### Supplementary Information

Supplementary Information.

## Data Availability

All data are provided in full in the results section and the Supplementary Information accompanying this paper.
