# Supplementary material for: Utility of oligonucleotide in upregulating circular RNA production in a cellular model
Source: Sci Rep. 2024 Apr 6;14:8096. doi: 10.1038/s41598-024-58663-x (PMC10998836; doi:10.1038/s41598-024-58663-x)
Supplement: Supplementary file 1 — Supplementary Information. [file 41598_2024_58663_MOESM1_ESM.pdf]

Supporting Information for

Utility of oligonucleotide in regulating circular RNA production in a cellular model

Lu Ni,[a] Takeshi Yamada,[a] Kazuhiko Nakatani[a]

[a] *Department of Regulatory Bioorganic Chemistry, SANKEN (The Institute of Scientific and Industrial Research), Osaka University, Mihogaoka 8-1, Ibaraki, Osaka 567-0047. JAPAN*

Table of Contents

Supplementary Figures ***3***
Figure S1. 4
Figure S2. 4
Figure S3. 4
Figure S4. 5
Figure S5. 5
Figure S6. 5
Figure S7. 6
Figure S8. 6
Figure S9. 6
Table S1 7

Supplementary Figures

A

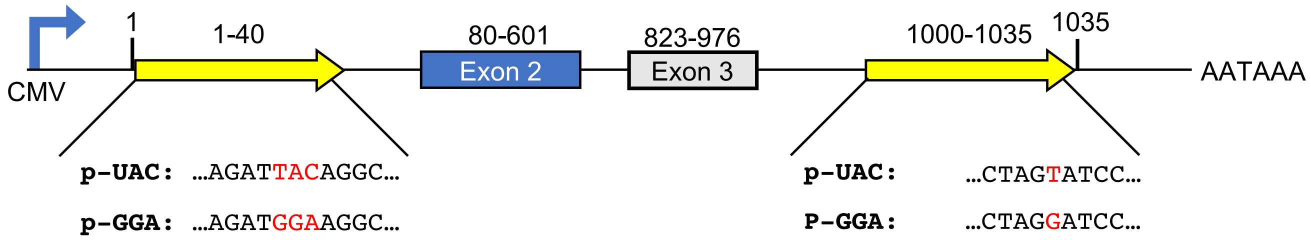

Plasmid	Resulting RNA transcript	Resulting circRNA
p-UAC	pre-mUAC	circZKSCAN1 containing a single nucleotide difference at 901
p-GGA	pre-mGGA	

B

TGGAATTCaaagtgctgagatTACaggcgtgagccaccacccccggccCACTTTTTGTAAAGGTACGTACTAATGACTTTTTTTTTATACTT
CAGGAATAGTAAAGAAACACATCATAAAACCTCCCAGGACATAAAGGTGAGCACAGACCCTGTTTGGATCAAGTCAGTTCCTGGAGCCTGAA
<-3′-TGGAGGGTCCTGTATTTCCACTC-5′: circZKSCAN1_Rev
TGATGACTGCTGAATCACGGGAAGCCACGGGTCTGTCCCCACAGGCTGCACAGGAGAAGGATGGTATCGTAATAGTGAAGGTGGAAGAGGAA
GATGAGGAAGACCACATGTGGGGGCAGGATTCCACCCTACAGGACACGCCTCCTCCAGACCCAGAGATATTCCGCCAACGCTTCAGGCGCTT
CTGTTACCAGAACACTTTTGGGCCCCGAGAGGCTCTCAGTCGGCTGAAGGAACTTTGTCATCAGTGGCTGCGGCCAGAAATAAACACCAAGG
AACAGATCCTGGAGCTTCTGGTGCTAGAGCAGTTTCTTTCCATCCTGCCCAAGGAGCTCCAGGTCTGGCTGCAGGAATACCGCCCCGATAGT
GGAGAGGAGGCCGTGACCCTTCTAGAAGACTTGGAGCTTGATTTATCAGGACAACAGGTAAAAAGAGGTGAAACCTATTATGTGTGAGCAGG
GCACAGACGTTGAAACTGGAGCCAGGAGAAGTATTGGCAGGCTTTAGGTTATTAGGTGGTTACTCTGTCTTAAAAATGTTCTGGCTTTCTTC
CTGCATCCACTGGCATACTCATGGTCTGTTTTTAAATATTTTAATTCCCATTTACAAAGTGATTTACCCACAAGCCCAACCTGTCTGTCTTC
circZKSCAN1_Fw: 5′-CAGTTCAGGAGTCCTCGAGCC-3′->
AGGTCCCAGGTCAAGTTCATGGACCTGAGATGCTCGCAAGGGGGATGGTGCCTCTGGATCCAGTTCAGGAGTCCTCGAGCCTTGACCTTCAT
CACGAGGCCACCCAGTCCCACTTCAAACATTCGTCTCGGAAACCCCGCCTCTTACAGTCACGAGGTAAGAAGCAAGGAAAAGAATTAggctc
ggcacggtagctcacacctGTAatcccagcaGCGGCCGCTCGAGTC

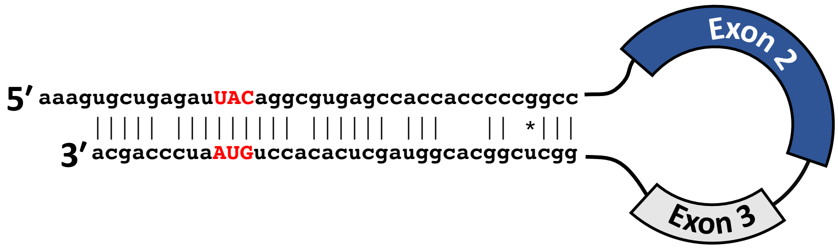

C

TGGAATTCaaagtgctgagatGGAaggcgtgagccaccacccccggccCACTTTTTGTAAAGGTACGTACTAATGACTTTTTTTTTATACTT
CAGGAATAGTAAAGAAACACATCATAAAACCTCCCAGGACATAAAGGTGAGCACAGACCCTGTTTGGATCAAGTCAGTTCCTGGAGCCTGAA
TGATGACTGCTGAATCACGGGAAGCCACGGGTCTGTCCCCACAGGCTGCACAGGAGAAGGATGGTATCGTAATAGTGAAGGTGGAAGAGGAA
GATGAGGAAGACCACATGTGGGGGCAGGATTCCACCCTACAGGACACGCCTCCTCCAGACCCAGAGATATTCCGCCAACGCTTCAGGCGCTT
CTGTTACCAGAACACTTTTGGGCCCCGAGAGGCTCTCAGTCGGCTGAAGGAACTTTGTCATCAGTGGCTGCGGCCAGAAATAAACACCAAGG
AACAGATCCTGGAGCTTCTGGTGCTAGAGCAGTTTCTTTCCATCCTGCCCAAGGAGCTCCAGGTCTGGCTGCAGGAATACCGCCCCGATAGT
GGAGAGGAGGCCGTGACCCTTCTAGAAGACTTGGAGCTTGATTTATCAGGACAACAGGTAAAAAGAGGTGAAACCTATTATGTGTGAGCAGG
GCACAGACGTTGAAACTGGAGCCAGGAGAAGTATTGGCAGGCTTTAGGTTATTAGGTGGTTACTCTGTCTTAAAAATGTTCTGGCTTTCTTC
CTGCATCCACTGGCATACTCATGGTCTGTTTTTAAATATTTTAATTCCCATTTACAAAGTGATTTACCCACAAGCCCAACCTGTCTGTCTTC
AGGTCCCAGGTCAAGTTCATGGACCTGAGATGCTCGCAAGGGGGATGGTGCCTCTGGATCCAGTTCAGGAGTCCTCGAGCCTTGACCTTCAT
CACGAGGCCACCCAGTCCCACTTCAAACATTCGTCTCGGAAACCCCGCCTCTTACAGTCACGAGGTAAGAAGCAAGGAAAAGAATTAggctc
ggcacggtagctcacacctGGAatcccagcaGCGGCCGCTCGAGTC

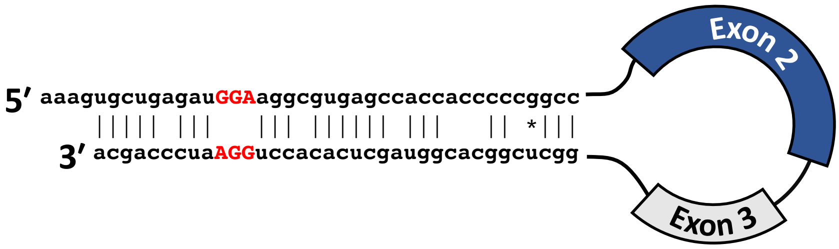

Figure S1. A) Specific mutation introduced to generate the **pre-mGGA** expressing construct **p-GGA**, from the original design, **p-UAC**, previously reported by Wilusz et al.,[1] the yellow arrows denote the RCMs in intronic sequences, the mutated nucleotides are highlighted in red, and the length of the sequences is denoted underneath individual elements. **B)** Whole sequence of the region coding for the original circZKSCAN1 in **p-UAC** (top), and the predicted resulting **pre-mUAC** hairpin structure (bottom). The divergent primer alignment is also shown, the arrow indicates the direction of PCR amplification. **C)** The whole sequence of the region coding for the **p-GGA** (top) and the predicted resulting **pre-mGGA** hairpin structure (bottom). Characters in blue denote the exon 2 region, and the ones highlighted in gray denote the exon 3 region. The yellow highlighted lower-case letters in bold denote the RCMs which are predicted to form the stem of the hairpin. Characters in bold underlined green denote the target sequences of CLIP-ON. The single nucleotide difference in exon 3 is highlighted in red, in the reference genome, it is expected to be T instead of C. The introduced mutations are highlighted in bold uppercase red characters.

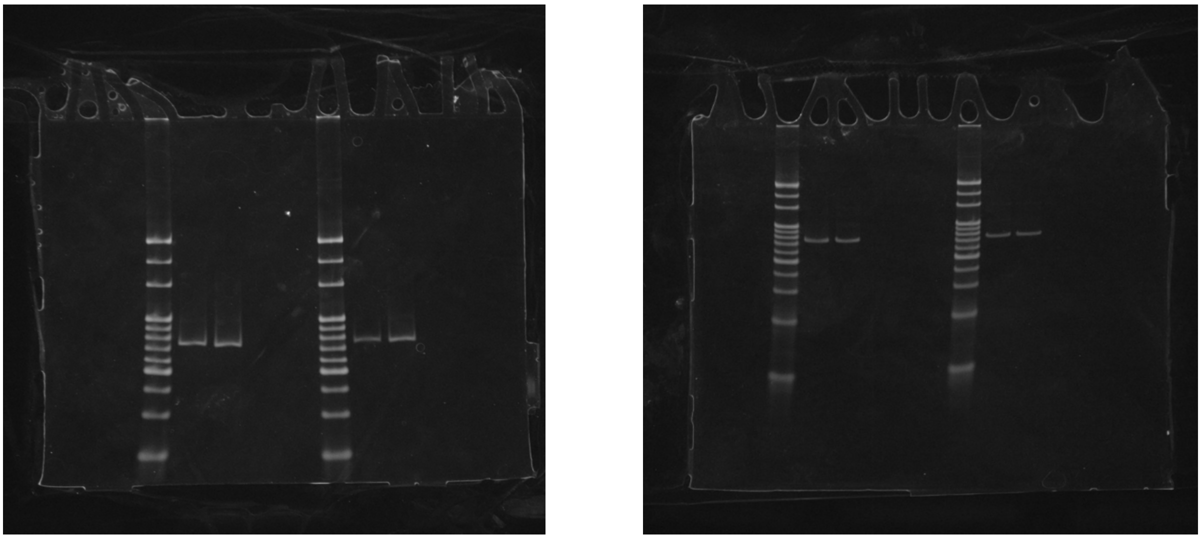

Figure S2. Left figure) The original gel file for Fig. 2C, shows a single band for both qPCR amplified products for pre-mUAC (grouped left of center) and pre-mGGA (grouped right of center). **Right Figure)** The original gel file for Fig. 2G, shows a single band of qPCR amplified products for pre-circEPHB4 (grouped right of center), additionally, due to the initial primer design resulting in the amplification of secondary products, the elimination of secondary products using the new primer set was checked with higher gel loading (grouped right of center).

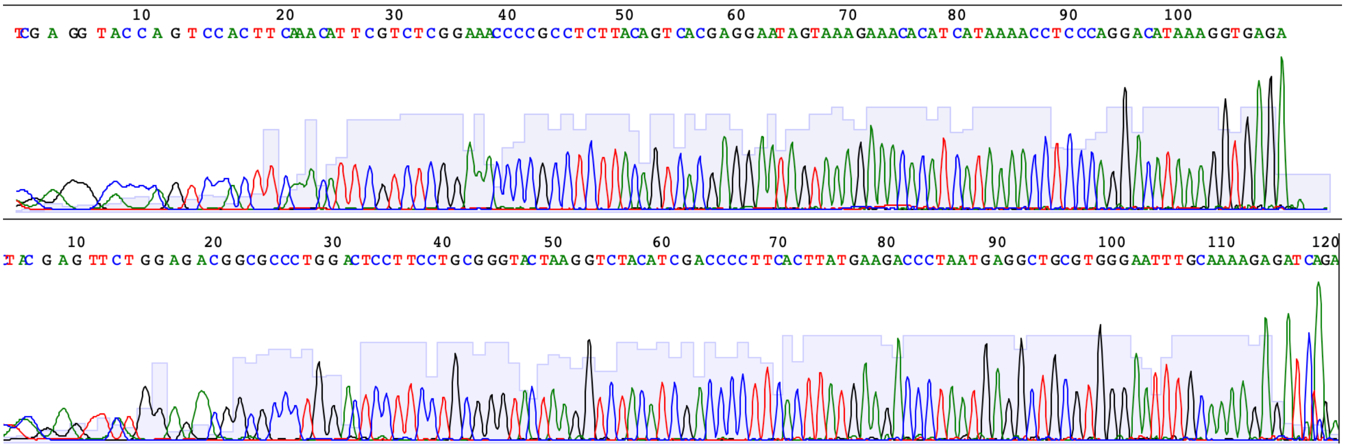

Figure S3. The Full sequence data of qPCR amplicon for circZKSCAN1 (top) and circEPHB4 (bottom).

TGGAATTCaaagtgctgagatTACaggcgtgagccaccacccccggccCACTTTTTGTAAAGGTACGTACTAATGACTTTTTTTTTATACTT
CAGGTACTAA

<-3′-GCACCCTTAAACGTTTTCTCTAG-5′: circEPHB4_Rev
GGTCTACATCGACCCCTTCACTTATGAAGACCCTAATGAGGCTGCGAGGGAATTTGCAAAAGAGATCGATGTCTCCTACGTCAAGATTGAAG
AGGTGATTGGTGCAGGTGAGAGCCGAAGGCTGCCCGGGCACCTGGGAACGAAGCGGGGGTGGGCAGGGCCACACTGGAGCGGGAGAGCTGAT
GACCTCTGCGTCCTTGTTTGAAGGTGAGTTTGGCGAGGTGTGCCGGGGGCGGCTCAAGGCCCCAGGGAAGAAGGAGAGCTGTGTGGCAATCA
AGACCCTGAAGGGTGGCTACACGGAGCGGCAGCGGCGTGAGTTTCTGAGCGAGGCCTCCATCATGGGCCAGTTCGAGCACCCCAATATCATC

5′-GCGTGGTCACCAACAGCAT-3′-> :circEPHB4_Fw
CGCCTGGAGGGCGTGGTCACCAACAGCATGCCCGTCATGATTCTCACAGAGTTCATGGAGAACGGCGCCCTGGACTCCTTCCTGCGGGTAAG
AAGCAAGGAAAAGAATTAggctcggcacggtagctcacacctGTAatcccagcagcgGCCGCTCGAGTC

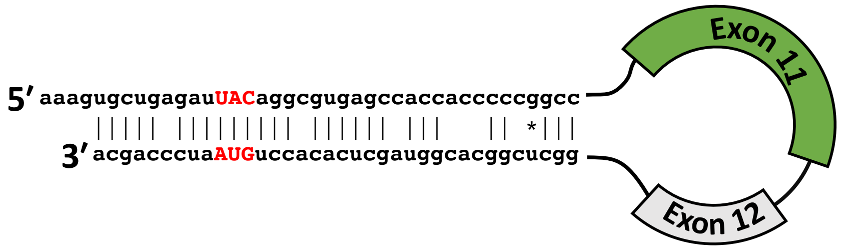

Figure S4. Whole sequence of the region coding for the circEPHB4 expressing p-circEPHB4, and the predicted resulting **pre-circEPHB4** hairpin structure (bottom). The divergent primer alignment is also shown and the arrow indicates the direction of PCR amplification. Characters in green denote the exon 11 region, and the ones highlighted in gray denote the exon 12 region. The yellow highlighted lower-case letters in bold denote the RCMs which are predicted to form the stem of the hairpin. Characters in bold underlined green denote the target sequences of CLIP-ON. The single nucleotide difference in exon 11 is highlighted in red, in the reference genome, it is expected to be T instead of C. A single mismatch within the designed circEPHB4_Rev primer is denoted to discriminate against another potential primer seed sequence located in exon 12 (exon 12, underlined sequence).

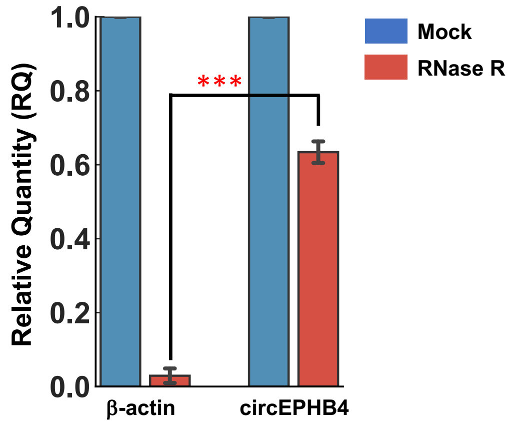

Figure S5. qPCR results following RNase R treatment of total RNA obtained from pre-circEPHB4 expressing HeLa cells (***: $p < 5 \times 10^{-4}$, two-tailed t-test, n=3)

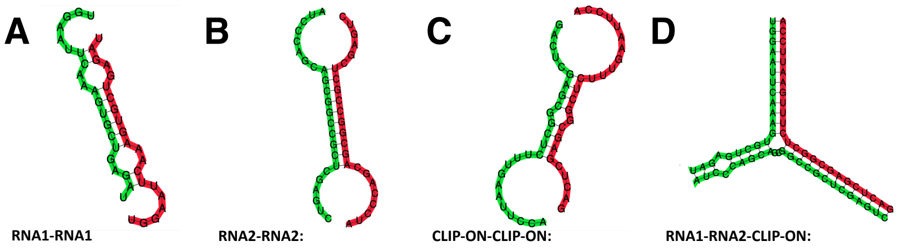

Figure S6. The visualized potential secondary structure of **A)** RNA1 dimer, **B)** RNA2 dimer, **C)** CLIP-ON dimer, and **D)** RNA1/RNA2/CLIP-ON complex.

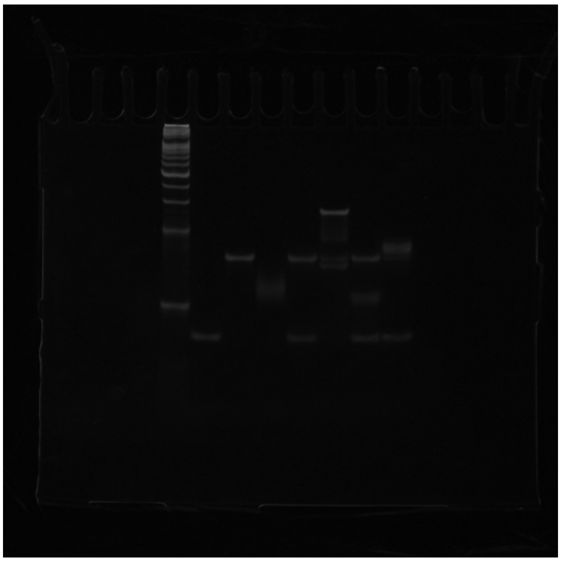

Figure S7. The original gel file for Fig. 3A., a native PAGE result of various RNA and/or ONs mixed under equimolar conditions.

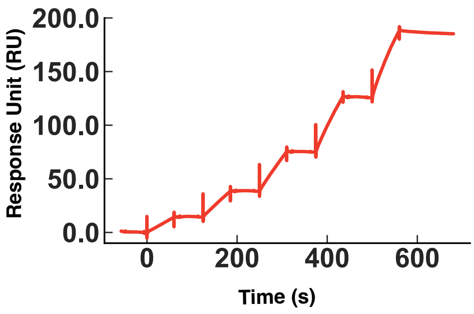

k_a	$1.53 \times 10^{4}\ M^{-1}$
k_d	$1.57 \times 10^{-4}\ s^{-1}$
K_D	$1.02 \times 10^{-8}\ M$

Figure S8. SPR assay to determine the affinity of **CLIP-ON** towards model hairpin RNA, b-TEG-RNA3 (Table S1), was immobilized on the gold surface containing its target sequence. Kinetic parameters of the binding of **CLIP-ON** to the RNA-immobilized surface were obtained using the single-cycle kinetics method and shown in the table on the left.

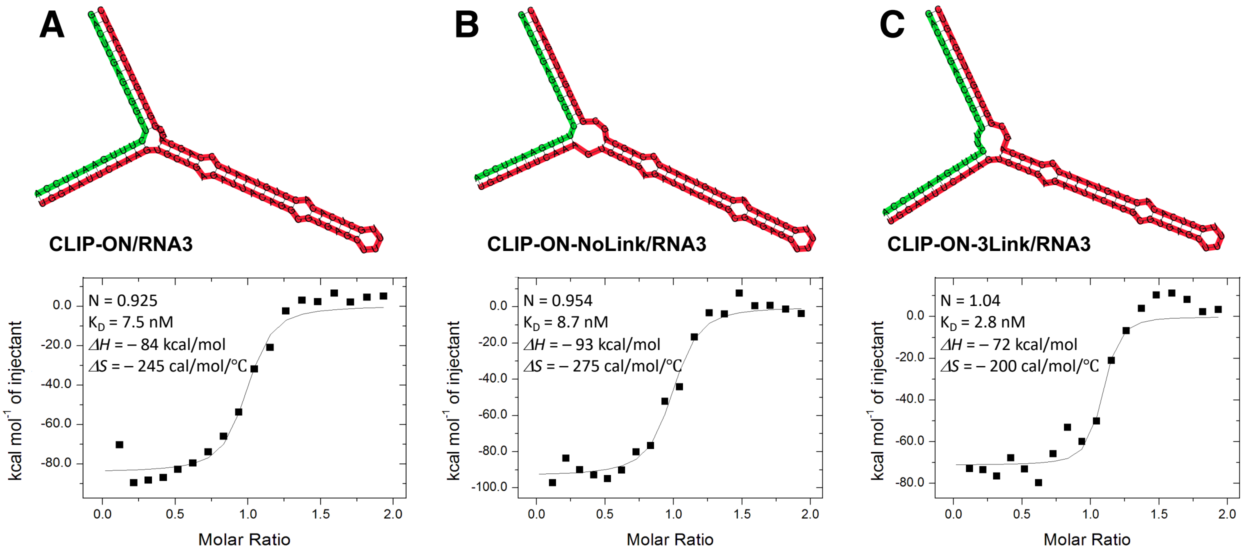

Figure S9. Visualization of the hybridized structure of **A)** CLIP-ON, **B)** CLIP-ON-NoLink, or **C)** CLIP-ON-3Link in green and the target RNA3 sequence in red, their respective ITC profiles are shown below.

Table S1. Primers and RNAs used for the experiments, the linker uracil is highlighted in bold red, CLIP-ON target sequences are highlighted in bold blue, and parital RCM sequence is highlighted in bold green

Sequence Name	Sequence
Beta_actin_mRNA_Fw	5′-CTCTTCCAGCCTTCCTTCCT-3′
Beta_actin_mRNA_Rev	5′-AGCACTGTGTTGGCGTACAG-3′
circZKSCAN1_Fw	5′-CAGTTCAGGAGTCCTCGAGCC-3′
circZKSCAN1_Rev	5′-CTCACCTTTATGTCCTGGGAGGT-3′
circEPHB4_Fw	5′-GCGTGGTCACCAACAGCAT-3′
circEPHB4_Rev	5′-GATCTCTTTTGCAAATTCCCACG-3′
RNA1	5′-**UGGAAUUCAAAGUGCUGAGAU**-3′
RNA2	5′-**AUCCCAGCA**GCG**GCCGCUCGAGUC**-3′
b-TEG-RNA3	Biotin-TEG-5′-**UGGAAUUCAAAGUGCUGAGAUUACAGGCGUGAGC UU**<br>**UU GCUCACACCUGUAAUCCCAGCA**GCG**GCCGCUCGAGUC**-3′
RNA3	5′-**UGGAAUUCAAAGUGCUGAGAUUACAGGCGUGAGC UU**<br>**UU GCUCACACCUGUAAUCCCAGCA**GCG**GCCGCUCGAGUC**-3′
